# Supplementary material for: Ephemeral-habitat colonization and neotropical species richness of Caenorhabditis nematodes
Source: BMC Ecol. 2017 Dec 19;17:43. doi: 10.1186/s12898-017-0150-z (PMC5738176; doi:10.1186/s12898-017-0150-z)

Additional File 7. Micro-habitat colonization and age-dependent incidence: Map of *Caenorhabditis* species recovery on baits (N=216) distributed at 72 spots along trail system (Parare, Nouragues Natural Reserve).

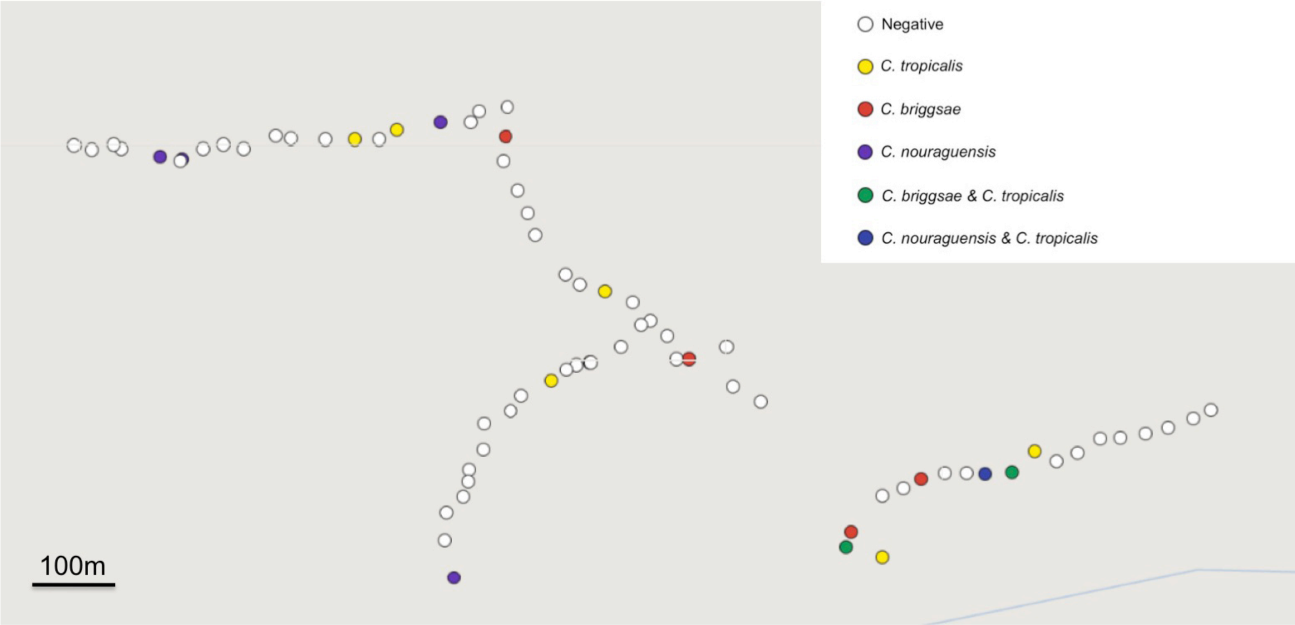

Supplement: Supplementary file 7 — Additional file 7. Micro-habitat colonization and age-dependent incidence: Map of Caenorhabditis species recovery on baits (N=216) distributed at 72 spots along trail system (Parare, Nouragues Natural Reserve). At each of the 72 spots (Parare, Nouragues Natural Reserve), three baits were distributed (i.e. subsamples, labelled a, b, c) approximately 1 meter apart from each other. Each sampling spot is indicated by a single dot, indicating species occurring in the three samples. At one site (#60), different samples contained C. briggsae and C. tropicalis. At another site (#59), a single subsample (59b) contained both C. nouraguensis and C. tropicalis. See Additional file 3 for detailed information. [file 12898_2017_150_MOESM7_ESM.pdf]
