# Supplementary material for: Ephemeral-habitat colonization and neotropical species richness of Caenorhabditis nematodes
Source: BMC Ecol. 2017 Dec 19;17:43. doi: 10.1186/s12898-017-0150-z (PMC5738176; doi:10.1186/s12898-017-0150-z)

Additional File 9. Map of opportunistic samples and *Caenorhabditis* species distribution at the Inselberg site (Nouragues Natural Reserve) (N=278).

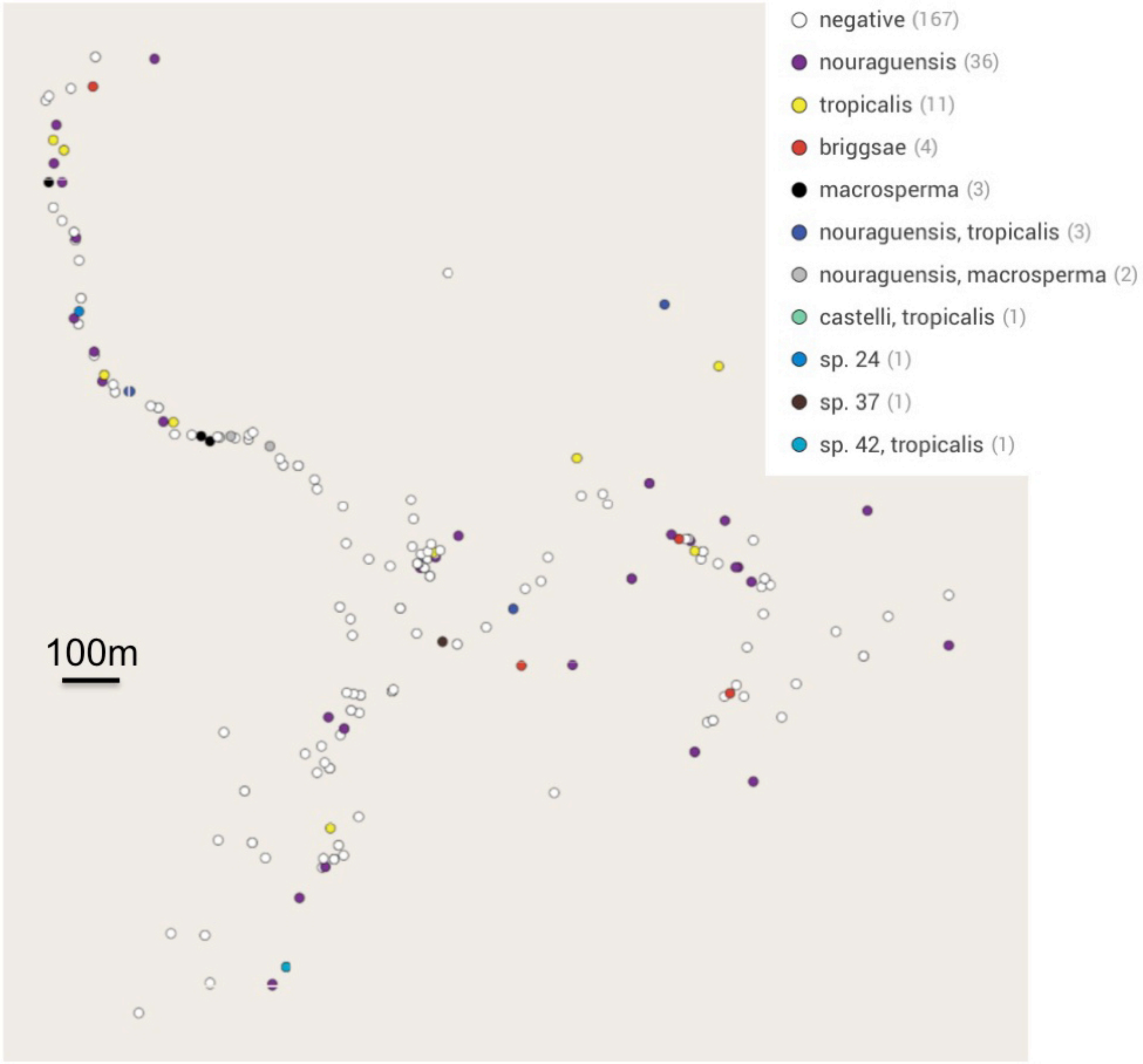

Supplement: Supplementary file 9 — Additional file 9. Map of opportunistic samples and Caenorhabditis species distribution at the Inselberg site (Nouragues Natural Reserve) (N=278). [file 12898_2017_150_MOESM9_ESM.pdf]
