# Supplementary material for: Ephemeral-habitat colonization and neotropical species richness of Caenorhabditis nematodes
Source: BMC Ecol. 2017 Dec 19;17:43. doi: 10.1186/s12898-017-0150-z (PMC5738176; doi:10.1186/s12898-017-0150-z)

Additional File 10. Map of opportunistic samples and *Caenorhabditis* species distribution at the Parare site (Nouragues Natural Reserve) (N=313).

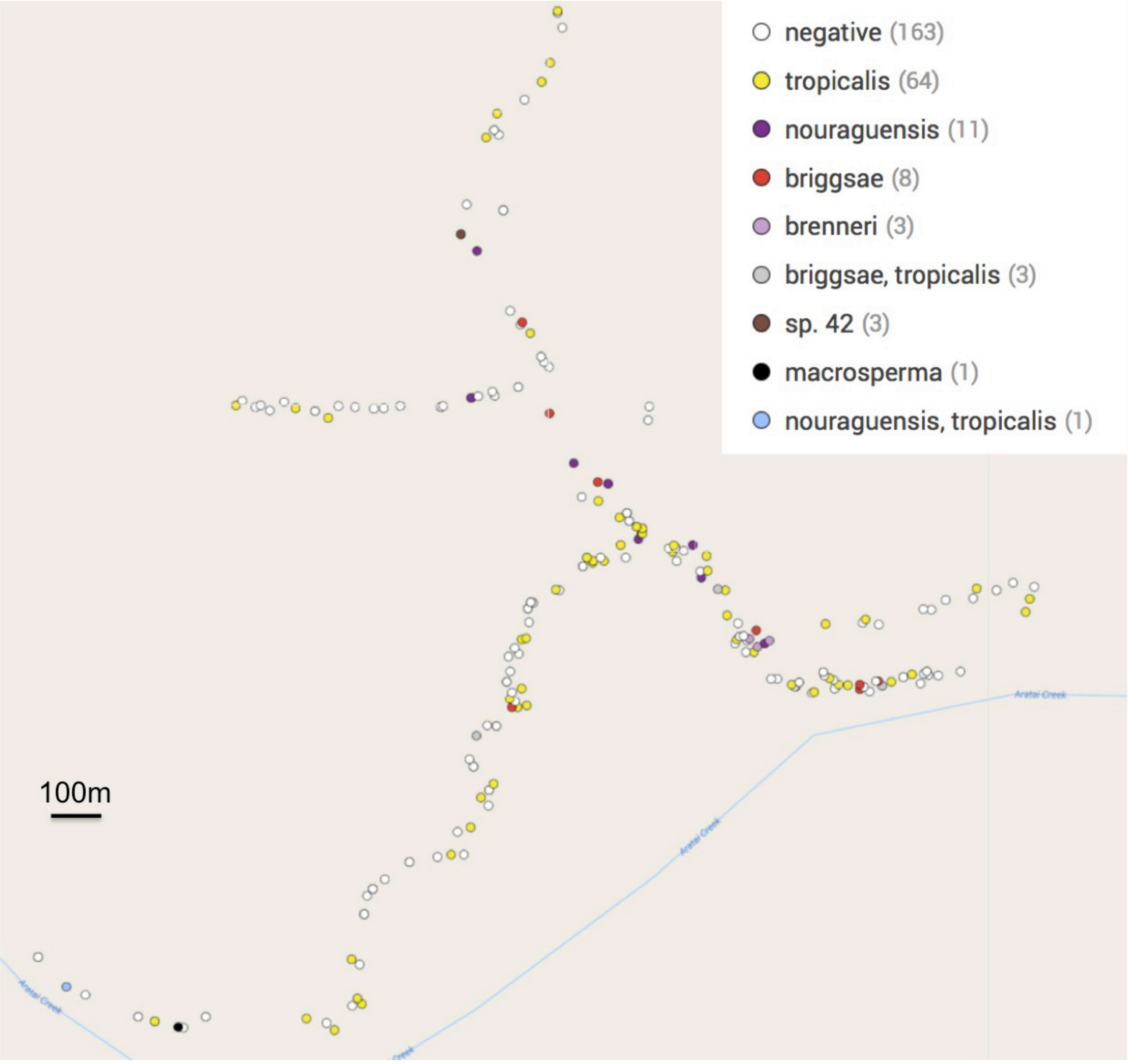

Supplement: Supplementary file 10 — Additional file 10. Map of opportunistic samples and Caenorhabditis species distribution at the Parare site (Nouragues Natural Reserve) (N=313). [file 12898_2017_150_MOESM10_ESM.pdf]
