# Supplementary material for: Ephemeral-habitat colonization and neotropical species richness of Caenorhabditis nematodes
Source: BMC Ecol. 2017 Dec 19;17:43. doi: 10.1186/s12898-017-0150-z (PMC5738176; doi:10.1186/s12898-017-0150-z)
Supplement: Supplementary file 12 — Additional file 12. Crosses of novel species (C. dolens, C. astrocarya) with known Caenorhabditis species [3]. Reciprocal crosses between different species and strains were performed as previously described [3], allowing for mating between 5–10 males and 5–10 females on a 55 mm diameter NGM plate. (A) Results of reciprocal crosses between the novel species C. dolens and presumptively close known relatives C. angaria, C. castelli, C. sp. 8, and C. astrocarya (B) Results of crosses between the novel species C. astrocarya and close known relatives, C. guadeloupensis and C. dolens. [file 12898_2017_150_MOESM12_ESM.pdf]

A) *C. dolens* NIC394

Cross 1: *C. dolens* (strain NIC394) x *C. angaria* (strain PS1010)

NIC394 male x PS1010 female:	no embyros	(4 replicates)
PS1010 male x NIC394 female:	no embryos	(3 replicates)

Cross 2: *C. dolens* (strain NIC394) x *C. castelli* (strain JU1426)

NIC394 male x JU1426 female:	dead embryos	(3 replicates)
JU1426 male x NIC394 female:	no embryos	(3 replicates)

Cross 3: *C. dolens* (strain NIC394) x *C. astrocarya* (strain NIC1040)

NIC394 male x NIC1040 female:	no embryos	(6 replicates)
NIC1040 male x NIC394 female:	few dead embryos	(7 replicates)

B) *C. astrocarya* NIC1040

Cross 1: *C. astrocarya* (strain NIC1040) x *C. guadeloupensis* (strain NIC113)

NIC1040 male x NIC113 female:	no embryos	(2 replicates)
NIC113 male x NIC1040 female:	no embryos	(2 replicates)

Cross 2: *C. astrocarya* (strain NIC1040) x *C. dolens* (strain NIC394)

NIC1040 male x NIC394 female:	few dead embryos	(7 replicates)
NIC394 male x NIC1040 female:	no embryos	(6 replicates)
