# Supplementary figures and images for: Ephemeral-habitat colonization and neotropical species richness of Caenorhabditis nematodes
Source: BMC Ecol. 2017 Dec 19;17:43. doi: 10.1186/s12898-017-0150-z (PMC5738176; doi:10.1186/s12898-017-0150-z)

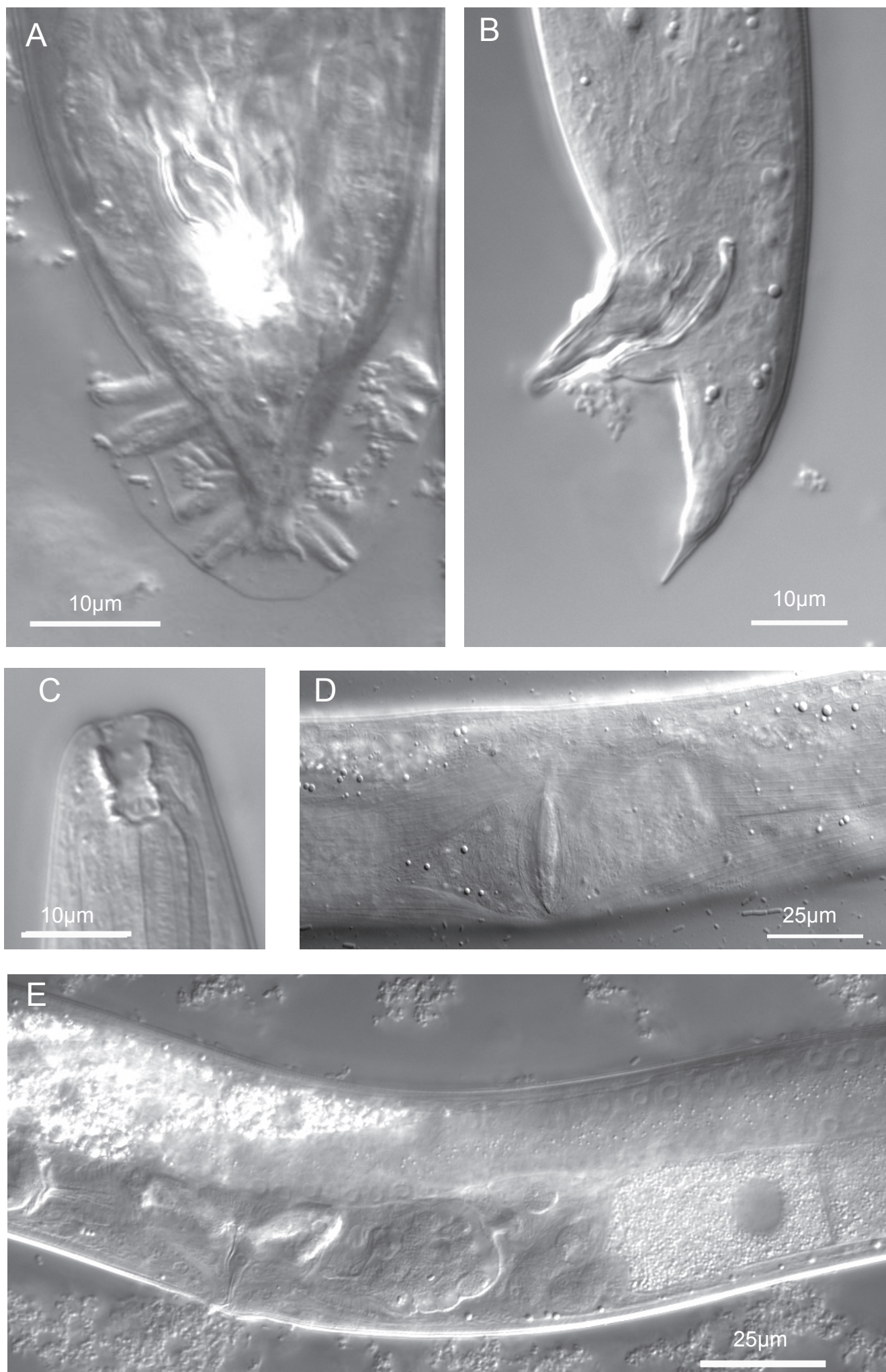

Supplement: Supplementary file 13 — Additional file 13. DIC microscopy images of C. dolens sp. n. (strain NIC394). (A) Ventral view of the adult male tail showing multiple ventral genital papillae. (B) Lateral view of adult male tail, focused on spicule. (C) Lateral view of buccal region (stoma) of adult female. (D) Ventral view of external vulval opening and cuticle of an adult female. (E) Lateral view of mid-body region of a young adult female showing vulva, uterus and germline (a maturing oocyte and spermatheca filled with sperm are visible). [file 12898_2017_150_MOESM13_ESM.pdf]

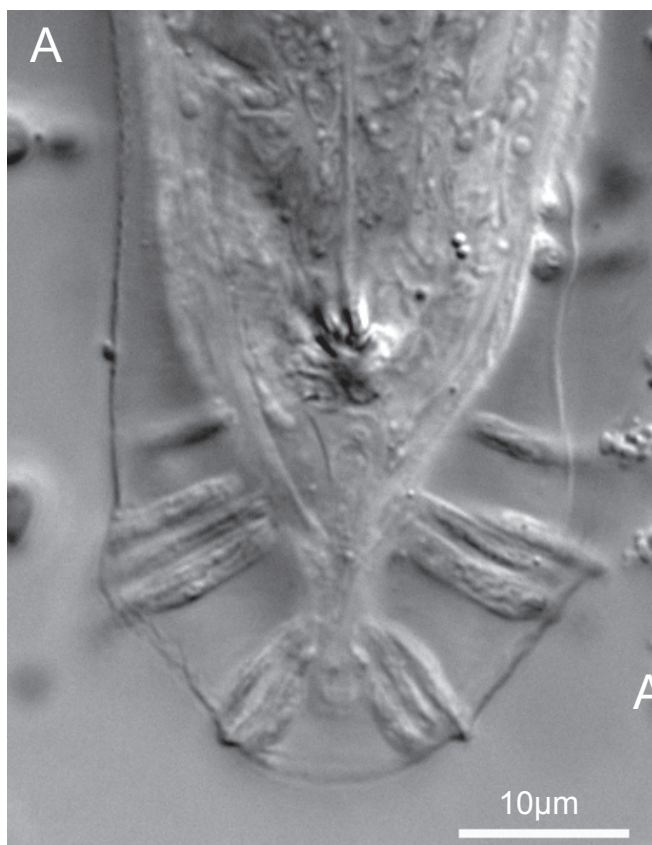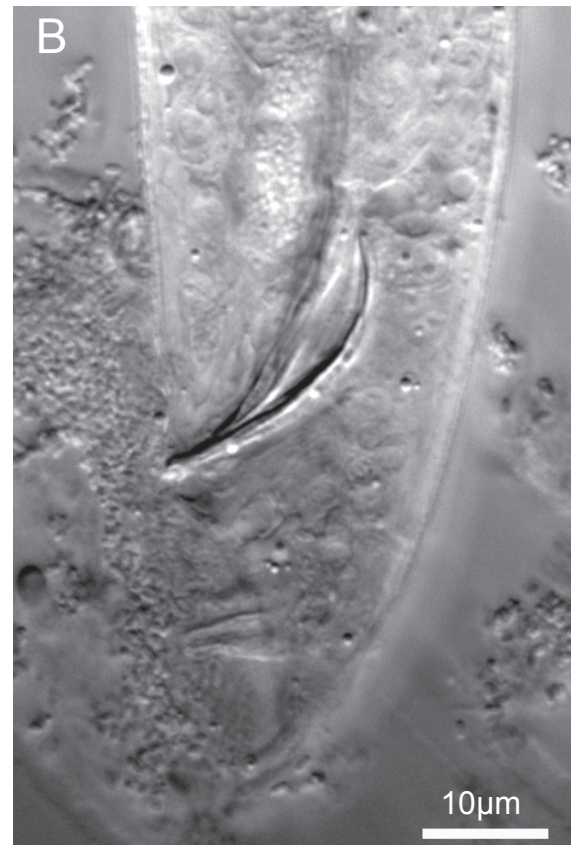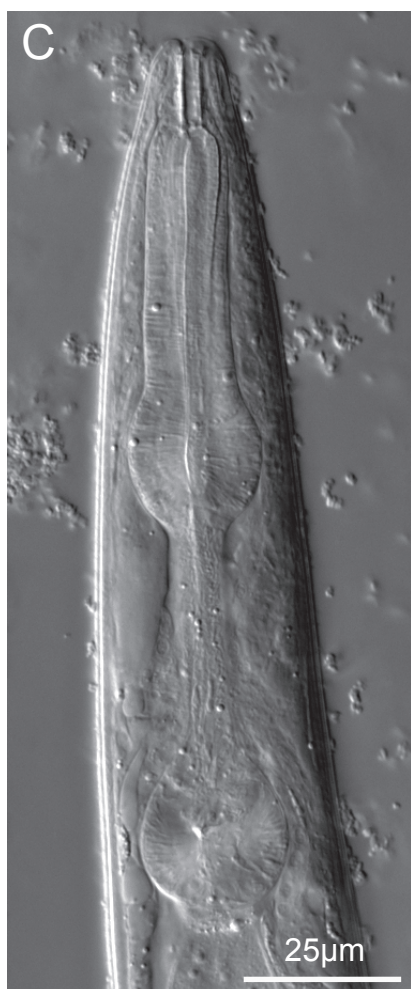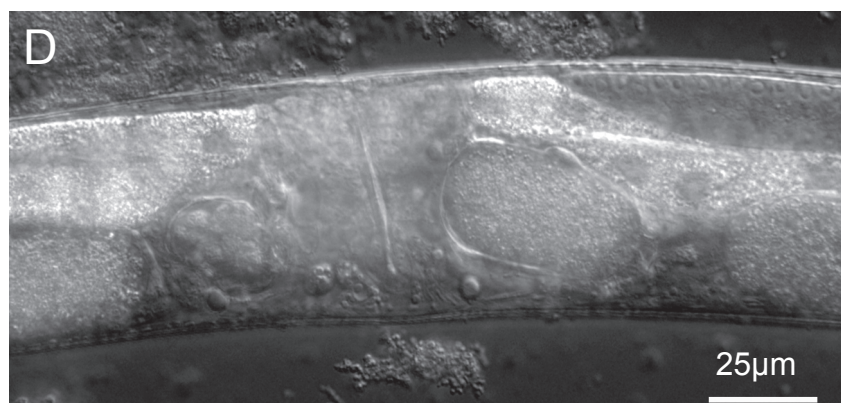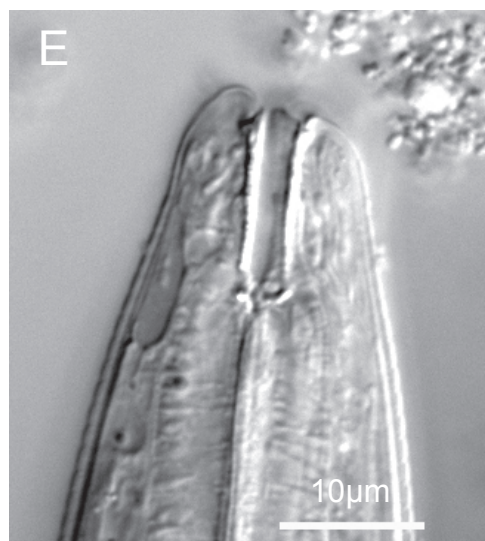

Supplement: Supplementary file 14 — Additional file 14. DIC microscopy images of C. astrocarya sp. n. (strain NIC1040). (A) Ventral view of the adult male tail showing multiple ventral and dorsal genital papillae. (B) Lateral view of adult male tail, focused on spicule, with several dorsal genital papillae visible. (C) Lateral view of pharynx region of an adult female. (D) Ventral view of mid-body region of any adult female showing vulva opening (to the right, two young embryos, and to the left, the spermatheca, are visible). (E) Lateral view of buccal region (stoma) of adult female. [file 12898_2017_150_MOESM14_ESM.pdf]
