# Supplementary material for: Ephemeral-habitat colonization and neotropical species richness of Caenorhabditis nematodes
Source: BMC Ecol. 2017 Dec 19;17:43. doi: 10.1186/s12898-017-0150-z (PMC5738176; doi:10.1186/s12898-017-0150-z)

**Additional File 15. Images of *Astrocaryum* palms (likely *Astrocaryum paramaca)* and inflorescence, representing the micro-habitat of the novel species, *C. astrocarya* (Nouragues Natural Reserve).**

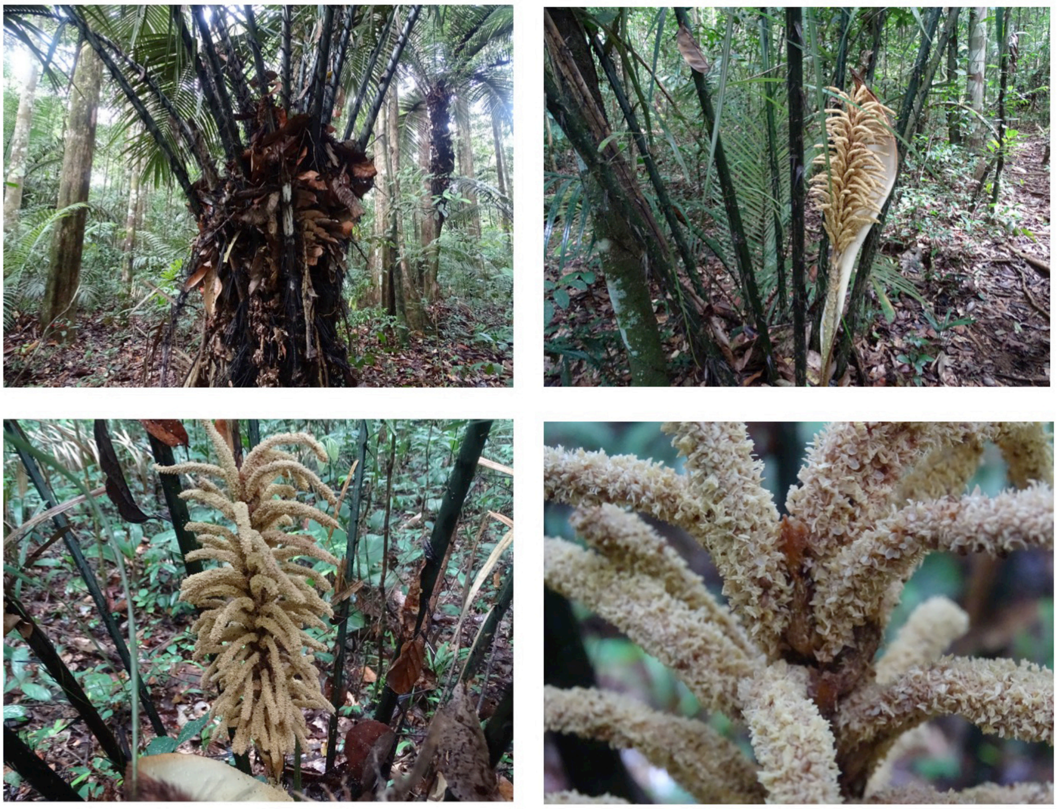

Supplement: Supplementary file 15 — Additional file 15. Images of Astrocaryum palms (likely Astrocaryum paramaca) and inflorescence, representing the micro-habitat of the novel species, C. astrocarya (Nouragues Natural Reserve). [file 12898_2017_150_MOESM15_ESM.pdf]
